# Prognostic implication of heart failure stage and left ventricular ejection fraction for patients with in-hospital cardiac arrest: a 16-year retrospective cohort study

**DOI:** 10.1007/s00392-024-02403-8

**Published:** 2024-02-26

**Authors:** Chih-Hung Wang, Li-Ting Ho, Meng-Che Wu, Cheng-Yi Wu, Joyce Tay, Pei-I. Su, Min-Shan Tsai, Yen-Wen Wu, Wei-Tien Chang, Chien-Hua Huang, Wen-Jone Chen

**Affiliations:** 1 Department of Emergency Medicine, National Taiwan University Hospital, No.7, Zhongshan S. Rd., Zhongzheng Dist., Taipei City 100, Taiwan Republic of China; 2 Department of Emergency Medicine, College of Medicine, National Taiwan University, Taipei, Taiwan; 3 Division of Cardiology, Department of Internal Medicine, National Taiwan University College of Medicine and Hospital, Taipei, Taiwan; 4 National Taiwan University College of Medicine and Hospital, Cardiovascular Center, Taipei, Taiwan; 5 Departments of Internal Medicine and Nuclear Medicine, National Taiwan University Hospital and National Taiwan University College of Medicine, Taipei, Taiwan; 6 Department of Nuclear Medicine and Cardiology Division of Cardiovascular Medical Center, Far Eastern Memorial Hospital, New Taipei City, Taiwan; 7 National Yang-Ming University School of Medicine, Taipei, Taiwan; 8 Department of Internal Medicine, Min-Sheng General Hospital, Taoyuan, Taiwan

**Keywords:** Heart failure, In-hospital cardiac arrest, Cardiopulmonary resuscitation, Neurological outcome, Survival

## Abstract

**Background:**

The 2022 AHA/ACC/HFSA guidelines for the management of heart failure (HF) makes therapeutic recommendations based on HF status. We investigated whether the prognosis of in-hospital cardiac arrest (IHCA) could be stratified by HF stage and left ventricular ejection fraction (LVEF).

**Methods:**

This single-center retrospective study analyzed the data of patients who experienced IHCA between 2005 and 2020. Based on admission diagnosis, past medical records, and pre-arrest echocardiography, patients were classified into general IHCA, at-risk for HF, pre-HF, HF with preserved ejection fraction (HFpEF), and HF with mildly reduced ejection fraction or HF with reduced ejection fraction (HFmrEF-or-HFrEF) groups.

**Results:**

This study included 2,466 patients, including 485 (19.7%), 546 (22.1%), 863 (35.0%), 342 (13.9%), and 230 (9.3%) patients with general IHCA, at-risk for HF, pre-HF, HFpEF, and HFmrEF-or-HFrEF, respectively. A total of 405 (16.4%) patients survived to hospital discharge, with 228 (9.2%) patients achieving favorable neurological recovery. Multivariable logistic regression analysis indicated that pre-HF and HFpEF were associated with better neurological (pre-HF, OR: 2.11, 95% confidence interval [CI]: 1.23–3.61, *p* = 0.006; HFpEF, OR: 1.90, 95% CI: 1.00–3.61, *p* = 0.05) and survival outcomes (pre-HF, OR: 2.00, 95% CI: 1.34–2.97, *p* < 0.001; HFpEF, OR: 1.91, 95% CI: 1.20–3.05, *p* = 0.007), compared with general IHCA.

**Conclusion:**

HF stage and LVEF could stratify patients with IHCA into different prognoses. Pre-HF and HFpEF were significantly associated with favorable neurological and survival outcomes after IHCA. Further studies are warranted to investigate whether HF status-directed management could improve IHCA outcomes.

**Supplementary Information:**

The online version contains supplementary material available at 10.1007/s00392-024-02403-8.

## Introduction

An estimated 292,000 patients in the United States experience in-hospital cardiac arrest (IHCA) each year [1]. Approximately 18.8% of patients with IHCA survive to hospital discharge, and only about 12.9% of these patients have favorable neurological recovery at hospital discharge [1].

Heart failure (HF) is a major public health issue, leading to over 1 million hospitalizations annually in the United States [2]. HF is present in approximately 20–30% of patients with IHCA [3–5], accounting for 12.6% of the etiologies of IHCA [6]. However, only a few studies [5, 7–10] have investigated the prognosis of HF complicated by IHCA. Also, the findings from these studies [5, 7–10] have been inconsistent and contradictory.

HF is a complex syndrome resulting from various functional or structural abnormalities impairing ventricular filling or blood pumping [11, 12]. The 2022 American Heart Association/American College of Cardiology/ Heart Failure Society of America (AHA/ACC/HFSA) Guidelines [11, 12] identify four stages to describe the trajectory of HF as a progressive disease. These stages include at-risk for HF (stage A), pre-HF (stage B), symptomatic HF (stage C), and advanced HF (stage D). In addition to HF stages, left ventricular ejection fraction (LVEF) is essential for HF management [11, 12].

The 2022 AHA/ACC/HFSA Guidelines [11, 12] make therapeutic recommendations based on HF stages and LVEF. Nevertheless, these recommendations focus on long-term management [11–13]. In the current study, we aimed to investigate the prognosis of IHCA stratified by HF stages and LVEF, which may facilitate the identification of patient groups susceptible to poor IHCA outcomes and tailoring of personalized management for patients with IHCA.

## Materials and Methods

This study was conducted in compliance with the Declaration of Helsinki amendments. The Research Ethics Committee of National Taiwan University Hospital (NTUH) approved this study (reference number: 201804089RINC) and waived the informed consent requirement. The study was conducted and reported according to the Strengthening the Reporting of Observational Studies in Epidemiology (STROBE) statement [14].

### Study design and setting

This observational study was a secondary analysis using prospectively collected IHCA databases registered in NTUH. NTUH is a tertiary medical center with 2,600 beds, including 220 intensive care unit (ICU) beds. The Centre of Quality Management at NTUH receives reports of all IHCA events and prospectively maintains an IHCA registry, aiming to improve patient safety systematically. When a patient experiences cardiac arrest in the general wards, a code team is activated per hospital policy. If cardiac arrest occurs in an ICU, resuscitation is performed by the staff in the unit without activating the code team. Cardiopulmonary resuscitation (CPR) is performed for all patients based on up-to-date resuscitation guidelines [15, 16].

### Patient inclusion criteria

For this study, the data of patients who experienced IHCA from 2005 to 2020 were reviewed and included, if they met the following criteria: (1) patients with age ≥ 18, (2) patients with a documented absence of pulse and record of receiving chest compressions for at least 2 min, and (3) patients without a do-not-resuscitate order prior to the arrest. Patients admitted for major trauma were excluded. In cases where a patient experienced multiple cardiac arrest events during their hospitalization, only the first event was retrieved for analysis.

### Data collection and variable definitions

Data regarding IHCA events were prospectively collected from the electronic medical records of NTUH. Patient comorbidities were defined according to Get With The Guidelines-Resuscitation registry [10, 17]. Peri-CPR variables were derived from the Utstein template [17]. In contrast, data used to reclassify patients by HF stages and LVEF for the current study were retrospectively collected, including N-terminal pro-B natriuretic peptide (NT-proBNP) and echocardiographic findings. Both NT-proBNP and echocardiographic findings were retrieved from the index hospitalization or within 3 months before the IHCA event [18]. Echocardiographic findings related to HF staging were abstracted [11, 12].

### Grouping by HF status

According to the 2022 AHA/ACC/HFSA Guidelines [11, 12], we classified HF into five groups based on the admission diagnosis, prior medical records, laboratory studies, and echocardiographic findings: (1) general IHCA, (2) at-risk for HF, (3) pre-HF, (4) HF with preserved ejection fraction (HFpEF), and (5) HF with mildly reduced EF or HF with reduced ejection fraction (HFmrEF-or-HFrEF). At-risk for HF was defined as the absence of HF in the admission diagnosis and prior medical records and the presence of hypertension, cardiovascular diseases, diabetes, or obesity [19]. Pre-HF was defined as the absence of HF in the admission diagnosis and prior medical records and the presence of structural heart disease, evidence of increased filling pressures, or elevated NT-proBNP level. The thresholds for echocardiographic parameters and NT-proBNP level to define pre-HF were consistent with the 2022 AHA/ACC/HFSA Guidelines [11, 12]. HFpEF, HFmrEF, and HFrEF were defined as the presence of HF in the admission diagnosis and/or prior medical records with LVEF $\ge$ 50%, between 41–49%, or ≤ 40% [11, 12], respectively. Patients who did not meet the above criteria were categorized as the general IHCA group.

### Study outcomes

The primary and secondary outcomes were favorable neurological status and survival at hospital discharge, respectively. Favorable neurological status was defined as a score of 1 or 2 on the Cerebral Performance Category (CPC) scale [20], which was retrospectively recorded by research assistants blinded to the research hypothesis.

### Statistical Analysis

Data were analyzed using R version 4.4.2 software (R Foundation for Statistical Computing, Vienna, Austria). Categorical data are expressed as counts and proportions, and continuous data are expressed as medians and interquartile ranges (IQRs). Categorical variables were compared using the chi-squared test, whereas continuous variables were compared using the Kruskal–Wallis test. A two-tailed* p*-value < 0.05 was considered statistically significant.

The odds ratio (OR) was selected as the outcome measure. Multivariable logistic regression analyses were used to examine the associations between independent variables and outcomes. All available independent variables, including HF status, age, sex, comorbidities, peri-CPR variables, and critical care interventions, were considered in the regression model, regardless of whether they were determined to be significant by univariate analyses. The stepwise variable selection procedure was adopted. Generalized additive models (GAMs) [21] were used to examine the nonlinear effects of the continuous variables and identify the appropriate cut-off point(s) for dichotomizing a continuous variable.

In the main analysis, the association between each HF status and outcomes was compared with that of general IHCA. Subgroup analysis was performed to examine the association between each HF status and IHCA period, diabetes, or initial arrest rhythms to identify the factors accounting for the differences in prognosis among the different HF categorizations. The IHCA period was split into three intervals based on the 2010 [22, 23] and 2015 [24, 25] guidelines for CPR. In the sensitivity analysis, only patients with available echocardiographic data were included. Echocardiographic parameters and NT-proBNP level were substituted for HF status in the regression analysis to examine the association between individual echocardiographic parameters with outcomes.

## Results

**Fig. 1:**
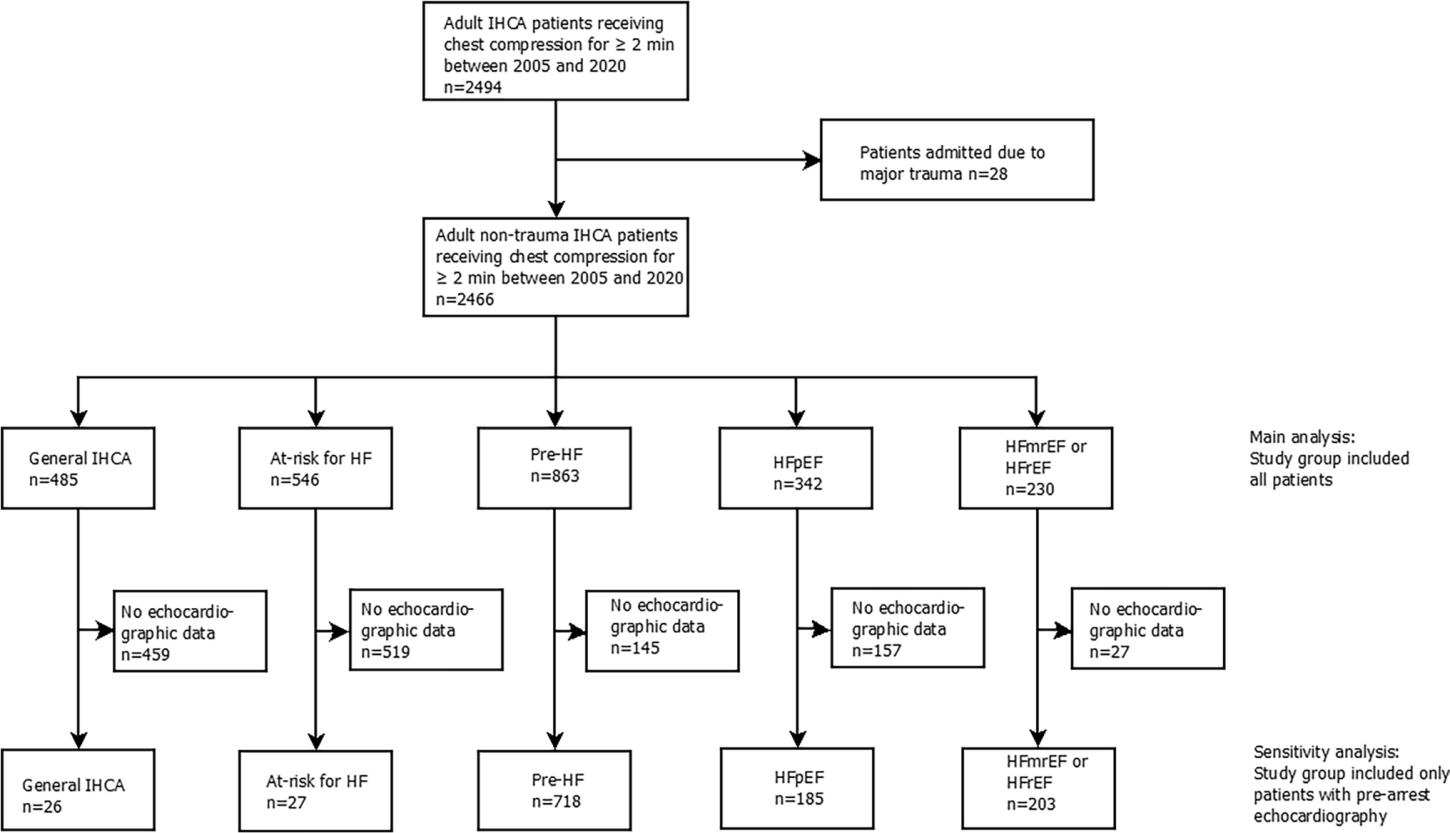
Patient selection flowchart. HF: heart failure; HFmrEF: heart failure with mildly reduced ejection fraction; HFpEF: heart failure with preserved ejection fraction; HFrEF: heart failure with reduced ejection fraction; IHCA: in-hospital cardiac arrest

**Fig. 2:**
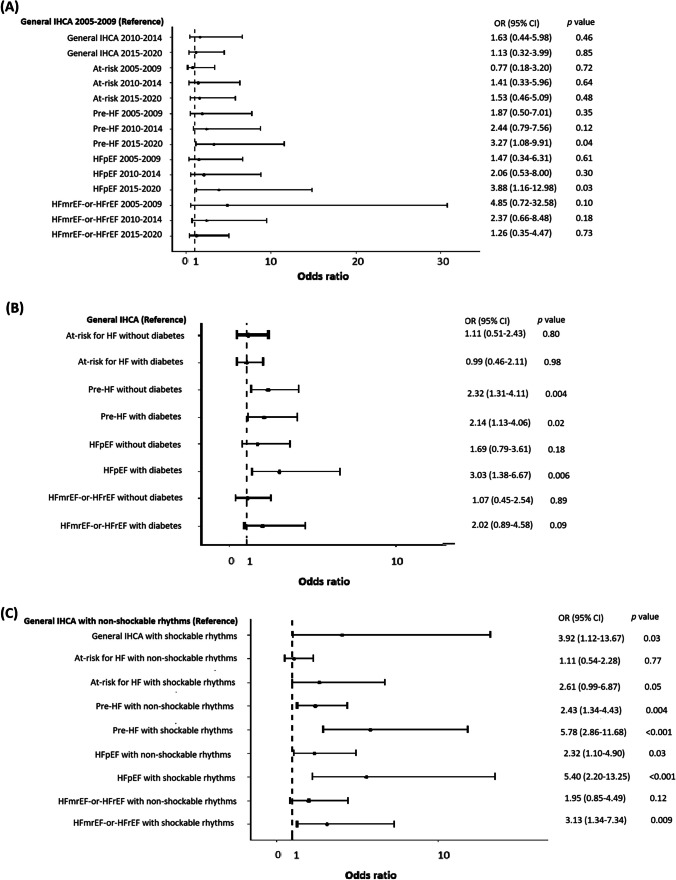
Forest plot of adjusted odds ratio of each subgroup for favorable neurological outcome at hospital discharge. Subgroups were stratified by HF status and (**A**) period of IHCA, (**B**) diabetes, and (**C**) initial arrest rhythms. Please refer to Supplemental Table 1–3 for the confounding factors accounted for. HF: heart failure; HFmrEF: heart failure with mildly reduced ejection fraction; HFpEF: heart failure with preserved ejection fraction; HFrEF: heart failure with reduced ejection fraction; IHCA: in-hospital cardiac arrest; OR: odds ratio

As shown in Figs. 1 and 2, 466 patients were included in the main analysis. Among these patients, 1,159 (47.0%) patients had echocardiographic data and were included in the sensitivity analysis.

In the main analysis (Table 1), 485 (19.7%), 546 (22.1%), 863 (35.0%), 342 (13.9%), and 230 (9.3%) patients were categorized as patients with general IHCA, at-risk for HF, pre-HF, HFpEF, and HFmrEF-or-HFrEF, respectively. Compared with patients with general IHCA, patients with at-risk of HF, pre-HF, HFpEF, or HFmrEF-or-HFrEF were older, had more comorbidity burdens except for metastatic cancer or any blood-borne malignancy, experienced higher proportions of initial shockable rhythms, and underwent more extracorporeal membrane oxygenation (ECMO) and percutaneous coronary intervention (PCI) procedures. A total of 405 (16.4%) patients survived to hospital discharge, with 228 (9.2%) patients achieving favorable neurological recovery. Patients with pre-HF, HFpEF, and HFmrEF-or-HFrEF had similar proportions of favorable neurological and survival outcomes, all of which were higher than that of general IHCA.

**Table 1:** Comparison of patients stratified by heart failure status in the main analysis

Variables	All patients (*n* = 2466)	General IHCA (*n* = 485)	At-risk for HF (*n* = 546)	Pre-HF (*n* = 863)	HFpEF (*n* = 342)	HFmrEF-or-HFrEF (*n* = 230)	*p*-value
Age, y (IQR)	67.6 (22.3)	59.2 (22.9)	69.3 (21.0)	68.2 (21.5)	73.7 (19.9)	71.6 (21.3)	< 0.001
Male, n (%)	1517 (61.5)	306 (63.1)	346 (63.4)	528 (61.2)	174 (50.9)	163 (70.9)	< 0.001
Period of IHCA, n (%)							< 0.001
2005–2009	642 (26.0)	183 (37.7)	204 (37.4)	140 (16.2)	96 (28.1)	19 (8.3)	
2010–2014	865 (35.1)	150 (30.9)	135 (24.7)	378 (43.8)	102 (29.8)	100 (43.5)	
2015–2020	959 (38.9)	152 (31.3)	207 (37.9)	345 (40.0)	144 (42.1)	111 (48.3)	
Comorbidities, *n* (%)							
Hypertension	942 (38.2)	0 (0)	321 (58.8)	341 (39.5)	174 (50.9)	106 (46.1)	< 0.001
Diabetes	861 (34.9)	0 (0)	277 (50.7)	317 (36.7)	153 (44.7)	114 (49.6)	< 0.001
Myocardial infarction, this admission	316 (12.8)	0 (0)	77 (14.1)	155 (18.0)	37 (10.8)	47 (20.4)	< 0.001
Myocardial infarction, prior admission	158 (6.4)	0 (0)	35 (6.4)	65 (7.5)	25 (7.3)	33 (14.3)	< 0.001
History of CABG	162 (6.5)	6 (1.2)	27 (4.9)	48 (5.6)	39 (11.4)	42 (18.3)	< 0.001
PAOD	90 (3.6)	0 (0)	12 (2.2)	38 (4.4)	18 (5.3)	22 (9.6)	< 0.001
Atherosclerotic cardiovascular disease	547 (22.2)	0 (0)	139 (25.5)	236 (27.3)	86 (25.1)	86 (37.4)	< 0.001
Arrhythmia	484 (19.6)	32 (6.6)	68 (12.5)	193 (22.4)	115 (33.6)	76 (33.0)	< 0.001
Pneumonia	770 (31.2)	159 (32.8)	151 (27.7)	265 (30.7)	134 (39.2)	61 (26.5)	0.003
COPD	133 (5.4)	16 (3.3)	25 (4.6)	45 (5.2)	27 (7.9)	20 (8.7)	0.007
Cirrhosis	158 (6.4)	42 (8.7)	49 (9.0)	46 (5.3)	13 (3.8)	8 (3.5)	< 0.001
Dialysis	426 (17.3)	43 (8.9)	84 (15.4)	153 (17.7)	78 (22.8)	68 (29.6)	< 0.001
Stroke	120 (4.9)	0 (0)	40 (7.3)	40 (4.6)	23 (6.7)	17 (7.4)	< 0.001
Baseline evidence of motor, cognitive, or functional deficits	778 (31.5)	113 (23.3)	173 (31.7)	297 (34.4)	121 (35.4)	74 (32.2)	< 0.001
Bacteremia	234 (9.5)	39 (8.0)	52 (9.5)	98 (11.4)	29 (8.5)	16 (7.0)	0.15
Metastatic cancer or any blood-borne malignancy	563 (22.8)	201 (41.4)	119 (21.8)	193 (22.4)	33 (9.6)	17 (7.4)	< 0.001
Obesity	341 (13.8)	0 (0)	112 (20.5)	139 (16.1)	54 (15.8)	36 (15.7)	< 0.001
Pre-arrest events, *n* (%)							
Hypotension	596 (24.2)	110 (22.7)	104 (19.0)	223 (25.8)	87 (25.4)	72 (31.3)	0.003
Respiratory insufficiency	1662 (67.4)	326 (67.2)	357 (65.4)	587 (68.0)	235 (68.7)	157 (68.3)	0.82
Renal insufficiency	985 (39.9)	121 (24.9)	199 (36.4)	354 (41.0)	178 (52.0)	133 (57.8)	< 0.001
Hepatic insufficiency	358 (14.5)	83 (17.1)	88 (16.1)	120 (13.9)	38 (11.1)	29 (12.6)	0.10
Metabolic or electrolyte abnormality	376 (15.2)	66 (13.6)	73 (13.4)	148 (17.1)	52 (15.2)	37 (16.1)	0.28
Favorable neurological status 24 h before cardiac arrest	1134 (46.0)	236 (48.7)	232 (42.5)	404 (46.8)	145 (42.4)	117 (50.9)	0.08
Peri-CPR conditions, *n* (%)							
Arrest at night	799 (32.4)	165 (34.0)	203 (37.2)	254 (29.4)	101 (29.5)	76 (33.0)	0.03
Arrest on weekend	531 (21.5)	110 (22.7)	127 (23.3)	188 (21.8)	68 (19.9)	38 (16.5)	0.25
Arrest location							0.001
Intensive care unit	1032 (41.8)	187 (38.6)	209 (38.3)	376 (43.6)	148 (43.3)	112 (48.7)	
General ward	1193 (48.4)	263 (54.2)	291 (53.3)	389 (45.1)	159 (46.5)	91 (39.6)	
Others	241 (9.8)	35 (7.2)	46 (8.4)	98 (11.4)	35 (10.2)	27 (11.7)	
Monitoring	1480 (60.0)	253 (52.2)	296 (54.2)	549 (63.6)	211 (61.7)	171 (74.3)	< 0.001
Witnessed arrest	1691 (68.6)	314 (64.7)	358 (65.6)	616 (71.4)	236 (69.0)	167 (72.6)	0.03
Shockable rhythms	421 (17.1)	37 (7.6)	78 (14.3)	157 (18.2)	71 (20.8)	78 (33.9)	< 0.001
Critical care interventions in place at time of arrest, *n* (%)							
Mechanical ventilation	638 (25.9)	120 (24.7)	144 (26.4)	227 (26.3)	91 (26.6)	56 (24.3)	0.93
Antiarrhythmics	448 (18.2)	63 (13.0)	102 (18.7)	167 (19.4)	70 (20.5)	46 (20.0)	0.02
Vasopressors	967 (39.2)	193 (39.8)	198 (36.3)	327 (37.9)	145 (42.4)	104 (45.2)	0.11
Dialysis	191 (7.7)	23 (4.7)	36 (6.6)	70 (8.1)	33 (9.6)	29 (12.6)	0.002
Pulmonary artery catheter	14 (0.6)	0 (0)	3 (0.5)	6 (0.7)	2 (0.6)	3 (1.3)	0.26
Intra-aortic balloon pumping	24 (1.0)	0 (0)	0 (0)	16 (1.9)	1 (0.3)	7 (3.0)	< 0.001
CPR duration, min (IQR)	25.0 (32.0)	27.0 (32.0)	29.5 (33.0)	21.0 (30.0)	24.0 (32.7)	23.0 (29.0)	0.002
Post-ROSC interventions, *n* (%)							
Extracorporeal membrane oxygenation	269 (10.9)	20 (4.1)	43 (7.9)	112 (13.0)	41 (12.0)	53 (23.0)	< 0.001
Therapeutic hypothermia	65 (2.6)	7 (1.4)	19 (3.5)	27 (3.1)	6 (1.8)	6 (2.6)	0.20
Percutaneous coronary intervention	112 (4.5)	2 (0.4)	17 (3.1)	57 (6.6)	12 (3.5)	24 (10.4)	< 0.001
Outcome, *n* (%)							
Favorable neurological outcome at hospital discharge	228 (9.2)	20 (4.1)	31 (5.7)	110 (12.7)	38 (11.1)	29 (12.6)	< 0.001
Survival to hospital discharge	405 (16.4)	38 (7.8)	62 (11.4)	187 (21.7)	71 (20.8)	47 (20.4)	< 0.001

CABG: Coronary artery bypass grafting; COPD: chronic obstructive pulmonary disease; CPR: cardiopulmonary resuscitation; HF: heart failure; HFmrEF: heart failure with mildly reduced ejection fraction; HFpEF: heart failure with preserved ejection fraction; HFrEF: heart failure with reduced ejection fraction; IHCA: in-hospital cardiac arrest; IQR: interquartile range; PAOD: peripheral arterial occlusive disease; ROSC: return of spontaneous circulation

In the main analysis, all independent variables were considered in the regression model for variable selection. After confounding factors were accounted for, the results (Table 2) indicated that pre-HF and HFpEF were associated with favorable neurological (pre-HF, OR: 2.11, 95% confidence interval [CI]: 1.23–3.61, *p* = 0.006; HFpEF, OR: 1.90, 95% CI: 1.00–3.61, *p* = 0.05) and survival outcomes (pre-HF, OR: 2.00, 95% CI: 1.34–2.97, *p* < 0.001; HFpEF, OR: 1.91, 95% CI: 1.20–3.05, *p* = 0.007).

**Table 2:** Multivariable logistic regression model for primary and secondary outcomes in the main analysis

Independent variable	Odds ratio	95% confidence interval	*p* value
Primary outcome: favorable neurological status at hospital discharge			
General IHCA patients	Reference		
At-risk for HF	0.98	0.52–1.85	0.94
Pre-HF	2.11	1.23–3.61	0.006
HFpEF	1.90	1.00–3.61	0.05
HFmrEF-or-HFrEF	1.33	0.67–2.63	0.41
Age between 30 and 75 (y)	2.01	1.39–2.90	< 0.001
Pneumonia	0.48	0.31–0.73	< 0.001
Cirrhosis	0.23	0.06–0.86	0.03
Baseline evidence of motor, cognitive, or functional deficits	0.38	0.25–0.57	< 0.001
Metastatic cancer or any blood borne malignancy	0.25	0.14–0.44	< 0.001
Obesity	0.58	0.35–0.96	0.04
Respiratory insufficiency	0.56	0.39–0.80	0.001
Hepatic insufficiency	0.46	0.23–0.92	0.03
Favorable neurological status 24 h before cardiac arrest	3.50	2.41–5.09	< 0.001
Arrest in locations other than intensive care unit or general ward	2.48	1.62–3.79	< 0.001
Monitoring	1.55	1.08–2.25	0.02
Shockable rhythm	2.35	1.62–3.40	< 0.001
Pulmonary artery catheter in place at time of arrest	7.53	1.77–31.98	0.006
CPR^a^ duration less than 20 (min)	8.12	5.50–11.98	< 0.001
Post-ROSC PCI	2.88	1.68–4.93	< 0.001
Secondary outcome: survival to hospital discharge			
General IHCA patients	Reference		
At-risk for HF	1.07	0.67–1.70	0.78
Pre-HF	2.00	1.34–2.97	< 0.001
HFpEF	1.91	1.20–3.05	0.007
HFmrEF-or-HFrEF	1.36	0.80–2.30	0.26
Age between 46 and 83 (y)	1.37	1.02–1.83	0.03
Cirrhosis	0.28	0.10–0.75	0.01
Baseline evidence of motor, cognitive, or functional deficits	0.71	0.54–0.95	0.02
Metastatic cancer or any blood borne malignancy	0.40	0.27–0.57	< 0.001
Hypotension	0.53	0.38–0.75	< 0.001
Respiratory insufficiency	0.64	0.49–0.84	0.001
Hepatic insufficiency	0.36	0.21–0.62	< 0.001
Favorable neurological status 24 h before cardiac arrest	1.68	1.30–2.19	< 0.001
Arrest in locations other than intensive care unit or general ward	1.91	1.34–2.71	< 0.001
Shockable rhythm	2.08	1.55–2.79	< 0.001
Pulmonary artery catheter in place at time of arrest	3.82	1.07–13.70	0.004
CPR duration less than 23 (min)	7.71	5.74–10.35	< 0.001
Post-ROSC PCI	3.04	1.88–4.92	< 0.001

CPR: cardiopulmonary resuscitation; HF: heart failure; HFmrEF: heart failure with mildly reduced ejection fraction; HFpEF: heart failure with preserved ejection fraction; HFrEF: heart failure with reduced ejection fraction; IHCA: in-hospital cardiac arrest; PCI: percutaneous coronary intervention; ROSC: return of spontaneous circulation

**Fig. 3:**
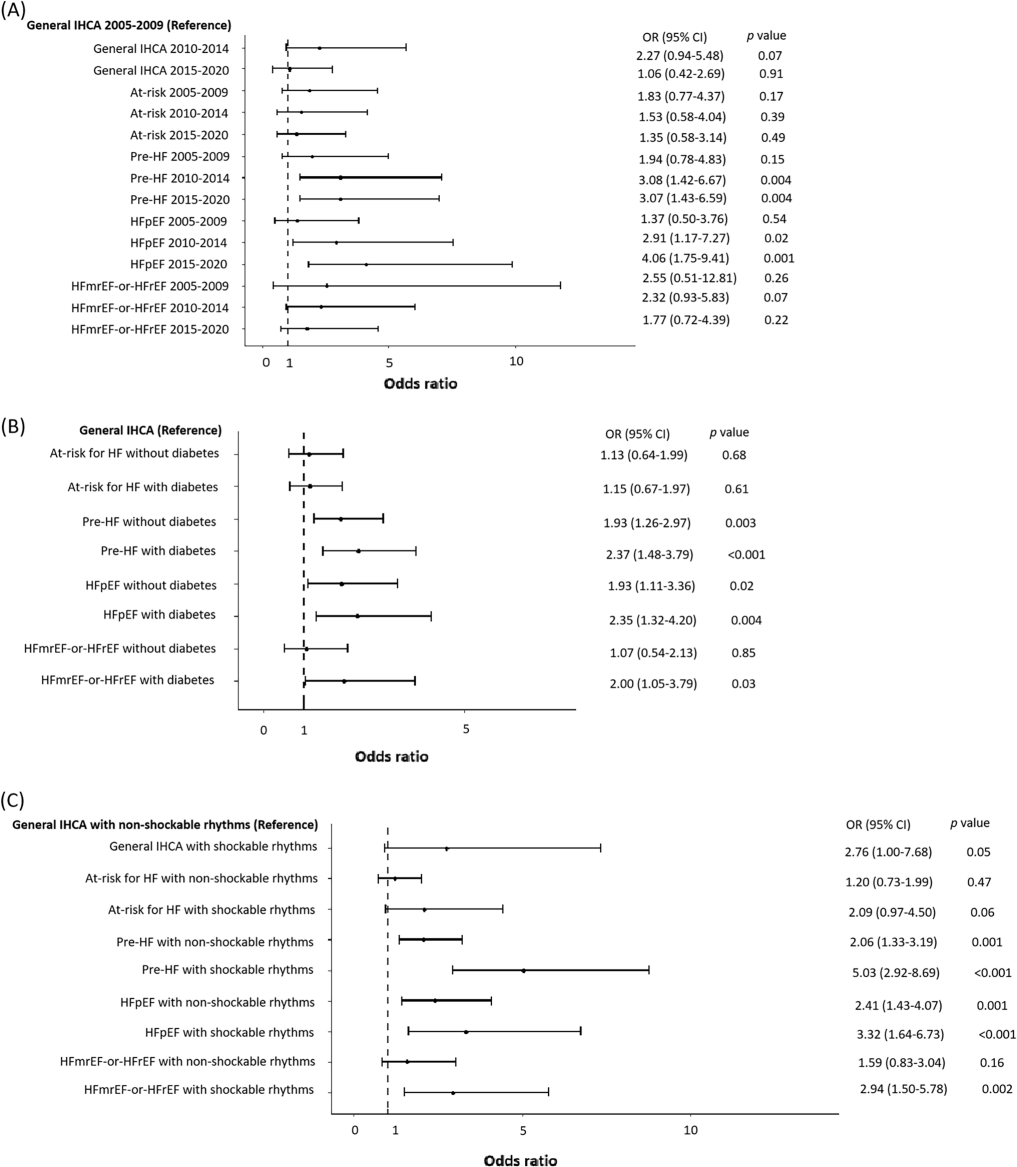
Forest plot of adjusted odds ratio of each subgroup for survival at hospital discharge. Subgroups were stratified by HF status and (**A**) period of IHCA, (**B**) diabetes, and (**C**) initial arrest rhythms. Please refer to Supplemental Table 1–3 for the confounding factors accounted for. HF: heart failure; HFmrEF: heart failure with mildly reduced ejection fraction; HFpEF: heart failure with preserved ejection fraction; HFrEF: heart failure with reduced ejection fraction; IHCA: in-hospital cardiac arrest; OR: odds ratio

The subgroup analysis (Figs. 2 and 3, Supplemental Table 1–3) revealed that during 2015–2020, pre-HF (OR: 3.27, 95% CI: 1.08–9.91, *p* = 0.04) and HFpEF (OR: 3.88, 95% CI: 1.16–12.98, *p* = 0.03) were associated with favorable neurological recovery. Additionally, for HF status stratified by diabetes or initial arrest rhythms, HFpEF with diabetes (OR: 3.03, 95% CI: 1.38–6.67, *p* = 0.006) and HFmrEF-or-HFrEF with shockable rhythms (OR: 3.12, 95% CI: 1.33–7.31, *p* = 0.009) were noted to be associated with favorable neurological outcomes.

Supplemental Table 4 shows the characteristics of patients who were included in the sensitivity analysis. The median time between pre-arrest echocardiography and IHCA event was 10.4 days (IQR: 50.9 days). Since not all patients had quantitative LVEF or NT-proBNP values, these variables were transformed into binary variables for analysis. Among all the echocardiographic parameters, only concentric remodeling was associated with favorable neurological recovery (OR: 1.66, 95% CI: 1.03–2.69, *p* = 0.04) (Supplemental Table 5).

## Discussion

### Main Findings

First, this 16-year cohort study revealed that pre-HF accounted for the highest proportion of IHCA. Second, the proportions of favorable neurological recovery in patients with pre-HF, HFpEF, or HFmrEF-or-HFrEF were similar, which were higher than that of general IHCA. Third, only pre-HF and HFpEF were significantly associated with better outcomes, which could be due to the presence of several subgroups stratified by HF status and IHCA period, diabetes, or initial shockable rhythms. Finally, among the echocardiographic parameters, only concentric remodeling was significantly associated with better outcomes.

### Comparison with previous studies

Hessulf et al. [5] analyzed the data of approximately 18,000 patients with IHCA during 2006–2015 and indicated that HF was significantly associated with lower survival. Using a large AHA registry, Chan et al. [10] analyzed the data of approximately 48,000 patients with IHCA during 2007–2010 and suggested that HF was not associated with survival. Considering LVEF, in a single-center study on 613 patients with IHCA during 2004–2006, Gonzalez et al. [18] suggested that LVEF < 45% was significantly associated with lower survival. Nevertheless, Aune et al. [7] suggested that LVEF < 50% was associated with higher survival by analyzing the data of 6,378 patients with IHCA during 2009–2019. Similarly, Chouairi et al. [8] studied 56,170 HF cases complicated with IHCA and demonstrated that, compared with HFpEF, HFrEF was significantly associated with higher survival after an IHCA event. Therefore, for all analyses of HF as an entire entity or stratified by LVEF, contradictory results were reported by the previous studies [5, 7–10].

### Grouping of patients with HF

In these previous studies [5, 7–10], patients with pre-HF were unidentified and thus mixed with patients with general IHCA for analysis. Our study revealed that, first, the proportion of pre-HF was the highest among all HF groups (Table 1). Second, approximately 21.9% of patients with pre-HF who had echocardiographic data demonstrated an LVEF < 50% (Supplemental Table 4). Third, pre-HF was associated with better outcomes (Table 2). Hence, the different methods in classifying pre-HF may account for the contradictory results of previous studies [5, 7–10]. For example, if pre-HF was analyzed as the absence of HF, the results may be biased toward favoring the positive association between the absence of HF and outcomes [5]. In contrast, if patients with pre-HF who had LVEF < 50% were counted as HFrEF, the results may suggest that HFrEF was associated with better outcomes [7, 8].

Besides at-risk for HF and pre-HF, no further classifications of symptomatic and advanced HF were made in our study. Advanced HF is defined as refractory symptoms despite optimal management [11, 12]. For advanced HF, palliative care is an important alternative. Most patients with advanced HF may not receive CPR if experiencing IHCA, leaving most patients with HF in our study classified as symptomatic HF and making further prognosis stratification unlikely. Therefore, LVEF was adopted to classify HF because LVEF is a critical factor in HF management [11, 12].

Table 1 demonstrates the significant differences in comorbidity burdens among these HF groups, with more cases of cardiovascular diseases in the HFmrEF-or-HFrEF group and more cases of cancer in the general IHCA group. These findings may explain the differences in the use of post-resuscitation interventions, such as ECMO and PCI. These significant between-group differences may justify our classification method for HF status.

### Main and subgroup analyses

Most previous studies [5, 7–10] investigated the association between HF and survival after an IHCA. Post–cardiac arrest myocardial dysfunction is an important component of post-cardiac arrest syndrome [26] and manifests as global hypokinesis, leading to reduced cardiac output and hypotension [26]. In a retrospective analysis, Yao et al. [27] indicated that HF was a significant predictor of post-cardiac arrest myocardial dysfunction. Therefore, patients with HF may be more likely to suffer from post-cardiac arrest hypotension caused by myocardial dysfunction. In our previous animal studies, we had noted the presence of prolonged post-cardiac arrest cerebral hypoperfusion [28–30], the so-called no-reflow phenomenon [31]. Post-cardiac arrest hypotension may worsen cerebral hypoperfusion, leading to worse neurological recovery [32, 33]. Taken together, patients with HF may have higher risk in developing post-cardiac arrest myocardial dysfunction, which may lead to post-arrest hypotension, aggravate cerebral hypoperfusion and worsen neurological recovery.

Our study was the first to reveal the neuroprognostic effect of different HF status on IHCA outcomes. Our study indicated that pre-HF or HFpEF was not only associated with survival but also with better neurological recovery than general IHCA. For pre-HF, no pharmacotherapy demonstrated definite benefits, except for patients with depressed LVEF [11, 12]. The natriuretic peptide-based screening for early identification of pre-HF was suggested by the 2017 ACC/AHA/HFSA update [34] to prevent the progression of pre-HF to HF. Jia et al. [35] indicated that incorporating biomarkers, such as NT-proBNP, could reclassify approximately 20% of older individuals without diagnosed HF to pre-HF. Interestingly, Table 1 demonstrates that the proportion of pre-HF among all patients with IHCA increased substantially after 2010. Possibly, since 2010, physicians initiated the use of biomarkers in evaluating patients with potential pre-HF more frequently. Identifying pre-HF early offers an opportunity to initiate lifestyle modification and better control of comorbidities, which may explain the better survival after 2010 and better neurological outcomes after 2015 for patients with pre-HF.

Similar to pre-HF, until now, HFpEF had no definitive benefits from therapy, except for sodium-glucose cotransporter-2 inhibitors [11, 12]. Nonetheless, during the study period, use of sodium-glucose cotransporter-2 inhibitors for HFpEF had not yet been approved in Taiwan. Among patients with HFpEF, 44.7% had diabetes (Table 1). Interestingly, the subgroup analysis revealed that patients with both HFpEF and diabetes were associated with favorable neurological recovery (Fig. 2). Jin et al. [36] indicated that in patients with diabetes, administration of metformin within 24 h before IHCA was associated with better neurological and survival outcomes, probably due to the cardioprotective [37] and neuroprotective effects [38–40] of metformin. According to the current American Diabetes Association guidelines [41], the effect of metformin on HF is considered neutral, and metformin is considered to be the first-line antidiabetic medication in all patients with type 2 diabetes, including those with HF. Therefore, most patients with HFpEF were possibly prescribed metformin in our study, leading to better outcomes.

Additionally, the subgroup analysis demonstrated that for patients with HFmrEF-or-HFrEF, the presence of shockable rhythms was associated with better outcomes (Figs. 2 and 3). For patients with HFmrEF-or-HFrEF, initial shockable rhythms may suggest the presence of a correctable cardiac cause, justifying the aggressive management of IHCA. In contrast, initial non-shockable rhythms may suggest that IHCA was caused by pump failure or by other non-cardiac causes, with HFmrEF-or-HFrEF being a significant comorbidity, impeding the success of the resuscitation efforts.

Taken together, the subgroup analysis helped explain some differences in prognosis among HF statuses. Nonetheless, due to reduced patient numbers in each subgroup, the results of subgroup analysis could only be considered hypothesis-generating or explanatory rather than definitive results.

### Sensitivity analysis

Few studies analyzed the pre-arrest echocardiographic data for IHCA. Using echocardiographic data measured within 3 months before IHCA, Gonzalez et al. [18] indicated that LVEF < 45% was associated with lower survival. Our sensitivity analysis indicated that only concentric remodeling was associated with better outcomes, whereas LVEF < 50% was not. Supplemental Table 4 shows that among patients with LVEF < 50%, 43.6% (157/360) were classified as patients with pre-HF. Since pre-HF was associated with better outcomes, the potential association between LVEF < 50% and worse outcomes may thus become less significant. Similarly, for patients with concentric remodeling, approximately 82.1% (230/280) were classified as patients with pre-HF, which may explain the significant association between concentric remodeling and favorable outcomes. These results suggest that, compared with individual echocardiographic findings, comprehensive HF status evaluated by HF stages and LVEF may be more prognostic of IHCA outcomes. This observation was consistent with that of a previous study [42], which demonstrated that isolated structural or functional abnormalities may not be associated with an increased risk of HF hospitalization or death. Nonetheless, due to the limitation of the retrospective study, most patients with general IHCA and at-risk for HF did not have recent echocardiographic data for comparison and were excluded from sensitivity analysis. This selection bias may distort the association between echocardiographic findings and IHCA outcomes.

### Future applications

The 2022 AHA/ACC/HFSA Guidelines for HF [11, 12] emphasized the importance of guideline-directed medical therapy delivered according to different HF statuses. Although CPR guidelines recommend that echocardiography should be performed early after the return of spontaneous circulation [15, 43], the application of these echocardiographic data to optimize post-CPR management remains unclear. Our study revealed that the prognosis of IHCA could be stratified by HF stages and LVEF. Future studies are warranted to investigate whether HF status-directed management, such as selection of hemodynamic goal, adequate administration of inotropic medications, and rehabilitation plans, could improve short- and long-term IHCA outcomes.

### Study Limitations

First, this study was a retrospective observational study, which could only establish an association between independent and dependent variables rather than a causal relationship. The current analysis did not consider critical confounding factors, such as administered medications. Second, instead of HF symptoms, the classification of HF groups was based on admission diagnosis or past medical records. Therefore, only patients with HF symptoms severe enough to be hospitalized would be categorized as HFpEF or HFmrEF-or-HFrEF. Otherwise, they would most likely be classified as pre-HF. Since symptoms were subjective and challenging to be studied retrospectively, further prospective studies are warranted. Third, HFmrEF was not separated from HFrEF because patients with HFmrEF usually remain in a dynamic trajectory, staggering between HFrEF and HFrEF. Also, no specific therapeutic recommendations were available exclusively for HFmrEF. Hence, patients with HFmrEF and HFrEF were combined to form one study group. Fourth, in comparison with 2022 AHA/ACC/HFSA Guidelines [11, 12], we did not use metabolic syndrome, exposure to cardiotoxic agents, genetic variant for cardiomyopathy, or positive family history of cardiomyopathy to define patients at risk for HF because these items were not required variables in the Get With The Guidelines-Resuscitation registry [10, 17]. Table 2 shows that there were no significant differences in neurological or survival outcomes between general IHCA and at-risk for HF. Misclassification of at-risk for HF may have occurred and caused the null results, which should be further examined in future studies.

## Conclusions

HF stage and LVEF could stratify patients with IHCA into different prognostic outcome groups. Pre-HF and HFpEF were significantly associated with favorable neurological recovery and survival after IHCA. Further studies are warranted to investigate whether HF status-directed management could improve IHCA outcomes.

## Supplementary Information

Below is the link to the electronic supplementary material.

Supplementary file1 (DOCX 55 KB)

## Data Availability

Data are available from the corresponding author upon reasonable request.
