# Supplementary material for: Prognostic implication of heart failure stage and left ventricular ejection fraction for patients with in-hospital cardiac arrest: a 16-year retrospective cohort study
Source: Clin Res Cardiol. 2024 Feb 26;114(5):557–69. doi: 10.1007/s00392-024-02403-8 (PMC12058836; doi:10.1007/s00392-024-02403-8)
Supplement: Supplementary file 1 — Supplementary file1 (DOCX 55 KB) [file 392_2024_2403_MOESM1_ESM.docx]

**Supplemental Table 1**. Subgroup analysis stratified by heart failure status and period of in-hospital cardiac arrest

| Independent variable | Odds ratio | 95% confidence interval | *p* value |
| --- | --- | --- | --- |
| *Primary outcome: favorable neurological status at hospital discharge* | | | |
| General IHCA 2005-2009 | Reference |  |  |
| General IHCA 2010-2014 | 1.63 | 0.44-5.98 | 0.46 |
| General IHCA 2015-2020 | 1.13 | 0.32-3.99 | 0.85 |
| At-risk 2005-2009 | 0.77 | 0.18-3.20 | 0.72 |
| At-risk 2010-2014 | 1.41 | 0.33-5.96 | 0.64 |
| At-risk 2015-2020 | 1.53 | 0.46-5.09 | 0.48 |
| Pre-HF 2005-2009 | 1.87 | 0.50-7.01 | 0.35 |
| Pre-HF 2010-2014 | 2.44 | 0.79-7.56 | 0.12 |
| Pre-HF 2015-2020 | 3.27 | 1.08-9.91 | 0.04 |
| HFpEF 2005-2009 | 1.47 | 0.34-6.31 | 0.61 |
| HFpEF 2010-2014 | 2.06 | 0.53-8.00 | 0.30 |
| HFpEF 2015-2020 | 3.88 | 1.16-12.98 | 0.03 |
| HFmrEF-or-HFrEF 2005-2009 | 4.85 | 0.72-32.58 | 0.10 |
| HFmrEF-or-HFrEF 2010-2014 | 2.37 | 0.66-8.48 | 0.18 |
| HFmrEF-or-HFrEF 2015-2020 | 1.26 | 0.35-4.47 | 0.73 |
| Age between 30 and 75 (y) | 1.98 | 1.37-2.86 | <0.001 |
| Pneumonia | 0.46 | 0.30-0.71 | <0.001 |
| Cirrhosis | 0.22 | 0.06-0.84 | 0.03 |
| Baseline evidence of motor, cognitive, or functional deficits | 0.40 | 0.26-0.60 | <0.001 |
| Metastatic cancer or any blood borne malignancy | 0.24 | 0.14-0.43 | <0.001 |
| Obesity | 0.53 | 0.32-0.89 | 0.02 |
| Respiratory insufficiency | 0.59 | 0.41-0.84 | 0.004 |
| Renal insufficiency | 0.69 | 0.48-1.00 | 0.05 |
| Favorable neurological status 24 h before cardiac arrest | 3.20 | 2.18-4.69 | <0.001 |
| Arrest in locations other than intensive care unit or general ward | 2.27 | 1.47-3.52 | <0.001 |
| Monitoring | 1.56 | 1.08-2.27 | 0.02 |
| Shockable rhythms | 2.40 | 1.64-3.50 | <0.001 |
| Pulmonary artery catheter in place at time of arrest | 8.16 | 1.94-34.32 | 0.004 |
| CPR duration less than 20 (min) | 8.64 | 5.78-12.92 | <0.001 |
| Post-ROSC PCI | 2.99 | 1.73-5.17 | <0.001 |
| *Secondary outcome: survival to hospital discharge* | | | |
| General IHCA 2005-2009 | Reference |  |  |
| General IHCA 2010-2014 | 2.27 | 0.94-5.48 | 0.07 |
| General IHCA 2015-2020 | 1.06 | 0.42-2.69 | 0.91 |
| At-risk 2005-2009 | 1.83 | 0.77-4.37 | 0.17 |
| At-risk 2010-2014 | 1.53 | 0.58-4.04 | 0.39 |
| At-risk 2015-2020 | 1.35 | 0.58-3.14 | 0.49 |
| Pre-HF 2005-2009 | 1.94 | 0.78-4.83 | 0.15 |
| Pre-HF 2010-2014 | 3.08 | 1.42-6.67 | 0.004 |
| Pre-HF 2015-2020 | 3.07 | 1.43-6.59 | 0.004 |
| HFpEF 2005-2009 | 1.37 | 0.50-3.76 | 0.54 |
| HFpEF 2010-2014 | 2.91 | 1.17-7.27 | 0.02 |
| HFpEF 2015-2020 | 4.06 | 1.75-9.41 | 0.001 |
| HFmrEF-or-HFrEF 2005-2009 | 2.55 | 0.51-12.81 | 0.26 |
| HFmrEF-or-HFrEF 2010-2014 | 2.32 | 0.93-5.83 | 0.07 |
| HFmrEF-or-HFrEF 2015-2020 | 1.77 | 0.72-4.39 | 0.22 |
| Age between 46 and 83 (y) | 1.40 | 1.05-1.88 | 0.02 |
| Cirrhosis | 0.27 | 0.10-0.74 | 0.01 |
| Baseline evidence of motor, cognitive, or functional deficits | 0.73 | 0.55-0.97 | 0.03 |
| Metastatic cancer or any blood borne malignancy | 0.39 | 0.27-0.56 | <0.001 |
| Hypotension | 0.54 | 0.38-0.76 | <0.001 |
| Respiratory insufficiency | 0.64 | 0.49-0.85 | 0.002 |
| Renal insufficiency | 0.75 | 0.57-0.98 | 0.04 |
| Hepatic insufficiency | 0.38 | 0.22-0.65 | <0.001 |
| Favorable neurological status 24 h before cardiac arrest | 1.58 | 1.20-2.06 | <0.001 |
| Arrest in locations other than intensive care unit or general ward | 1.95 | 1.36-2.79 | <0.001 |
| Shockable rhythms | 2.13 | 1.58-2.87 | <0.001 |
| Pulmonary artery catheter in place at time of arrest | 3.98 | 1.10-14.34 | 0.03 |
| CPR duration less than 23 (min) | 7.89 | 5.85-10.65 | <0.001 |
| Post-ROSC PCI | 2.97 | 1.82-4.84 | <0.001 |

CPR: cardiopulmonary resuscitation; HF: heart failure; HFmrEF: heart failure with mildly reduced ejection fraction; HFpEF: heart failure with preserved ejection fraction; HFrEF: heart failure with reduced ejection fraction; IHCA: in-hospital cardiac arrest; PCI: percutaneous coronary intervention; ROSC: return of spontaneous circulation

**Supplemental Table 2.** Subgroup analysis stratified by heart failure status and diabetes

| Independent variable | Odds ratio | 95% confidence interval | *p* value |
| --- | --- | --- | --- |
| *Primary outcome: favorable neurological status at hospital discharge* | | | |
| General IHCA | Reference |  |  |
| At-risk for HF without diabetes | 1.11 | 0.51-2.43 | 0.80 |
| At-risk for HF with diabetes | 0.99 | 0.46-2.11 | 0.98 |
| Pre-HF without diabetes | 2.32 | 1.31-4.11 | 0.004 |
| Pre-HF with diabetes | 2.14 | 1.13-4.06 | 0.02 |
| HFpEF without diabetes | 1.69 | 0.79-3.61 | 0.18 |
| HFpEF with diabetes | 3.03 | 1.38-6.67 | 0.006 |
| HFmrEF-or-HFrEF without diabetes | 1.07 | 0.45-2.54 | 0.89 |
| HFmrEF-or-HFrEF with diabetes | 2.02 | 0.89-4.58 | 0.09 |
| Age between 30 and 75 (y) | 2.00 | 1.39-2.88 | <0.001 |
| Pneumonia | 0.46 | 0.30-0.71 | <0.001 |
| Cirrhosis | 0.23 | 0.06-0.86 | 0.03 |
| Baseline evidence of motor, cognitive, or functional deficits | 0.38 | 0.25-0.58 | <0.001 |
| Metastatic cancer or any blood borne malignancy | 0.24 | 0.14-0.43 | <0.001 |
| Obesity | 0.54 | 0.32-0.90 | 0.02 |
| Respiratory insufficiency | 0.60 | 0.42-0.86 | 0.005 |
| Renal insufficiency | 0.67 | 0.47-0.97 | 0.04 |
| Hepatic insufficiency | 0.50 | 0.25-0.99 | 0.05 |
| Favorable neurological status 24 h before cardiac arrest | 3.44 | 235-5.02 | <0.001 |
| Arrest in locations other than intensive care unit or general ward | 2.41 | 1.57-3.70 | <0.001 |
| Monitoring | 1.53 | 1.06-2.22 | 0.02 |
| Shockable rhythms | 2.35 | 1.61-3.41 | <0.001 |
| Pulmonary artery catheter in place at time of arrest | 8.23 | 1.98-34.31 | 0.004 |
| CPR duration less than 20 (min) | 8.82 | 5.92-13.16 | <0.001 |
| Post-ROSC PCI | 2.84 | 1.65-4.89 | <0.001 |
| *Secondary outcome: survival to hospital discharge* | | | |
| General IHCA | Reference |  |  |
| At-risk for HF without diabetes | 1.13 | 0.64-1.99 | 0.68 |
| At-risk for HF with diabetes | 1.15 | 0.67-1.97 | 0.61 |
| Pre-HF without diabetes | 1.93 | 1.26-2.97 | 0.003 |
| Pre-HF with diabetes | 2.37 | 1.48-3.79 | <0.001 |
| HFpEF without diabetes | 1.93 | 1.11-3.36 | 0.02 |
| HFpEF with diabetes | 2.35 | 1.32-4.20 | 0.004 |
| HFmrEF-or-HFrEF without diabetes | 1.07 | 0.54-2.13 | 0.85 |
| HFmrEF-or-HFrEF with diabetes | 2.00 | 1.05-3.79 | 0.03 |
| Cirrhosis | 0.26 | 0.10-0.70 | 0.008 |
| Baseline evidence of motor, cognitive, or functional deficits | 0.72 | 0.54-0.95 | 0.02 |
| Metastatic cancer or any blood borne malignancy | 0.39 | 0.27-0.56 | <0.001 |
| Hypotension | 0.53 | 0.37-0.75 | <0.001 |
| Respiratory insufficiency | 0.66 | 0.51-0.87 | 0.003 |
| Renal insufficiency | 0.73 | 0.56-0.96 | 0.02 |
| Hepatic insufficiency | 0.39 | 0.23-0.67 | <0.001 |
| Favorable neurological status 24 h before cardiac arrest | 1.65 | 1.27-2.15 | <0.001 |
| Arrest in locations other than intensive care unit or general ward | 1.85 | 1.30-2.63 | <0.001 |
| Shockable rhythms | 2.00 | 1.48-2.69 | <0.001 |
| Pulmonary artery catheter in place at time of arrest | 3.78 | 1.06-13.53 | 0.04 |
| CPR duration less than 23 (min) | 8.26 | 6.11-11.18 | <0.001 |
| Post-ROSC PCI | 2.73 | 1.67-4.46 | <0.001 |

CPR: cardiopulmonary resuscitation; HF: heart failure; HFmrEF: heart failure with mildly reduced ejection fraction; HFpEF: heart failure with preserved ejection fraction; HFrEF: heart failure with reduced ejection fraction; IHCA: in-hospital cardiac arrest; PCI: percutaneous coronary intervention; ROSC: return of spontaneous circulation

**Supplemental Table 3**. Subgroup analysis stratified by heart failure status and initial arrest rhythms

| Independent variable | Odds ratio | 95% confidence interval | *p* value |
| --- | --- | --- | --- |
| *Primary outcome: favorable neurological status at hospital discharge* | | | |
| General IHCA with non-shockable rhythms | Reference |  |  |
| General IHCA with shockable rhythms | 3.92 | 1.12-13.67 | 0.03 |
| At-risk for HF with non-shockable rhythms | 1.11 | 0.54-2.28 | 0.77 |
| At-risk for HF with shockable rhythms | 2.61 | 0.99-6.87 | 0.05 |
| Pre-HF with non-shockable rhythms | 2.43 | 1.34-4.43 | 0.004 |
| Pre-HF with shockable rhythms | 5.78 | 2.86-11.68 | <0.001 |
| HFpEF with non-shockable rhythms | 2.32 | 1.10-4.90 | 0.03 |
| HFpEF with shockable rhythms | 5.40 | 2.20-13.25 | <0.001 |
| HFmrEF-or-HFrEF with non-shockable rhythms | 1.95 | 0.85-4.49 | 0.12 |
| HFmrEF-or-HFrEF with shockable rhythms | 3.13 | 1.34-7.34 | 0.009 |
| Age between 30 and 75 (y) | 2.02 | 1.40-2.92 | <0.001 |
| Pneumonia | 0.47 | 0.30-0.72 | <0.001 |
| Cirrhosis | 0.24 | 0.06-0.89 | 0.03 |
| Baseline evidence of motor, cognitive, or functional deficits | 0.39 | 0.26-0.60 | <0.001 |
| Metastatic cancer or any blood borne malignancy | 0.24 | 0.14-0.43 | <0.001 |
| Obesity | 0.57 | 0.34-0.94 | 0.03 |
| Respiratory insufficiency | 0.58 | 0.41-0.83 | 0.003 |
| Renal insufficiency | 0.68 | 0.48-0.98 | 0.04 |
| Hepatic insufficiency | 0.47 | 0.24-0.95 | 0.04 |
| Favorable neurological status 24 h before cardiac arrest | 3.32 | 2.28-4.85 | <0.001 |
| Arrest in locations other than intensive care unit or general ward | 2.45 | 1.59-3.75 | <0.001 |
| Monitoring | 1.51 | 1.04-2.19 | 0.03 |
| Pulmonary artery catheter in place at time of arrest | 7.97 | 1.92-32.97 | 0.004 |
| CPR duration less than 20 (min) | 8.36 | 5.65-12.38 | <0.001 |
| Post-ROSC PCI | 2.87 | 1.67-4.93 | <0.001 |
| *Secondary outcome: survival to hospital discharge* | | | |
| General IHCA with non-shockable rhythms | Reference |  |  |
| General IHCA with shockable rhythms | 2.76 | 1.00-7.68 | 0.05 |
| At-risk for HF with non-shockable rhythms | 1.20 | 0.73-1.99 | 0.47 |
| At-risk for HF with shockable rhythms | 2.09 | 0.97-4.50 | 0.06 |
| Pre-HF with non-shockable rhythms | 2.06 | 1.33-3.19 | 0.001 |
| Pre-HF with shockable rhythms | 5.03 | 2.92-8.69 | <0.001 |
| HFpEF with non-shockable rhythms | 2.41 | 1.43-4.07 | 0.001 |
| HFpEF with shockable rhythms | 3.32 | 1.64-6.73 | <0.001 |
| HFmrEF-or-HFrEF with non-shockable rhythms | 1.59 | 0.83-3.04 | 0.16 |
| HFmrEF-or-HFrEF with shockable rhythms | 2.94 | 1.50-5.78 | 0.002 |
| Age between 46 and 83 (y) | 1.38 | 1.03-1.84 | 0.03 |
| Cirrhosis | 0.26 | 0.10-0.71 | 0.009 |
| Baseline evidence of motor, cognitive, or functional deficits | 0.73 | 0.55-0.97 | 0.03 |
| Metastatic cancer or any blood borne malignancy | 0.39 | 0.27-0.56 | <0.001 |
| Hypotension | 0.52 | 0.36-0.73 | <0.001 |
| Respiratory insufficiency | 0.66 | 0.51-0.87 | 0.003 |
| Renal insufficiency | 0.74 | 0.57-0.97 | 0.03 |
| Hepatic insufficiency | 0.38 | 0.22-0.65 | <0.001 |
| Favorable neurological status 24 h before cardiac arrest | 1.63 | 1.26-2.13 | <0.001 |
| Arrest in locations other than intensive care unit or general ward | 1.89 | 1.32-2.68 | <0.001 |
| Pulmonary artery catheter in place at time of arrest | 3.93 | 1.11-13.93 | 0.03 |
| CPR duration less than 23 (min) | 8.26 | 6.10-11.17 | <0.001 |
| Post-ROSC PCI | 2.82 | 1.73-4.60 | <0.001 |

CPR: cardiopulmonary resuscitation; HF: heart failure; HFmrEF: heart failure with mildly reduced ejection fraction; HFpEF: heart failure with preserved ejection fraction; HFrEF: heart failure with reduced ejection fraction; IHCA: in-hospital cardiac arrest; PCI: percutaneous coronary intervention; ROSC: return of spontaneous circulation

**Supplemental Table 4**. Comparison of the patients stratified by heart failure status in the sensitivity analysis

| Variables | All patients  (n = 1159) | General IHCA (n = 26) | At-risk for HF (n = 27) | Pre-HF (n = 718) | HFpEF (n = 185) | HFmrEF-or-HFrEF (n = 203) | *p*-value |
| --- | --- | --- | --- | --- | --- | --- | --- |
| Age, y (IQR) | 69.5 (21.1) | 55.3 (19.5) | 67.2 (25.5) | 68.4 (21.3) | 73.6 (19.9) | 70.9 (21.3) | <0.001 |
| Male, n (%) | 711 (61.3) | 15 (57.7) | 21 (77.8) | 441 (61.4) | 93 (50.3) | 141 (69.5) | 0.001 |
| Period of IHCA, n (%) |  |  |  |  |  |  | <0.001 |
| 2005–2009 | 233 (20.1) | 11 (42.3) | 18 (66.7) | 136 (18.9) | 50 (27.0) | 18 (8.9) |  |
| 2010–2014 | 517 (44.6) | 7 (26.9) | 8 (29.6) | 330 (46.0) | 72 (38.9) | 100 (49.3) |  |
| 2015–2020 | 409 (35.3) | 8 (30.8) | 1 (3.7) | 252 (35.1) | 63 (34.1) | 85 (41.9) |  |
| Laboratory or echocardiographic findings, n (%) |  |  |  |  |  |  |  |
| LVMI >115 (males) or >95 (females) (g/m^2^) | 522 (45.0) | 0 (0) | 0 (0) | 286 (39.8) | 79 (42.7) | 157 (77.3) | <0.001 |
| Relative wall thickness >0.42 | 580 (50.0) | 0 (0) | 0 (0) | 426 (59.3) | 87 (47.0) | 67 (33.0) | <0.001 |
| Left ventricular wall thickness $\geq$12 (mm) | 362 (31.2) | 0 (0) | 0 (0) | 235 (32.7) | 53 (28.6) | 74 (36.5) | <0.001 |
| LVEF <50 (%) | 360 (31.1) | 0 (0) | 0 (0) | 157 (21.9) | 0 (0) | 203 (100) | <0.001 |
| LVEF, % (IQR) | 57.2 (27.3) (n=935) | 66.0 (11.2) (n=12) | 64.9 (9.8) (n=12) | 62.0 (21.8) (n=586) | 62.5 (13.3) (n=123) | 33.6 (15.9) (n=202) | <0.001 |
| E/e’ $\geq$15 | 244 (21.1) | 0 (0) | 0 (0) | 164 (22.8) | 37 (20.0) | 43 (21.2) | 0.004 |
| TR velocity >2.8 (m/s) | 427 (36.8) | 0 (0) | 0 (0) | 285 (39.7) | 66 (35.7) | 76 (37.4) | <0.001 |
| NT-proBNP $\geq$125 (pg/ml) | 231 (19.9) | 0 (0) | 0 (0) | 114 (15.9) | 57 (30.8) | 60 (29.6) | <0.001 |
| NT-proBNP, pg/ml (IQR) | 10871.0 (32105.0) (n=394) | 485.0 (0)  (n=1) | 2695.4 (991.0) (n=2) | 7130.0 (17957.0) (n=203) | 12814.5 (29412.5) (n=74) | 23400.0 (27845.5) (n=114) | <0.001 |
| Pattern of left ventricular remodeling, n (%) |  |  |  |  |  |  | <0.001 |
| Normal geometry | 368 (31.8) | 26 (100) | 27 (100) | 210 (29.2) | 78 (42.2) | 27 (13.3) |  |
| Concentric remodeling | 280 (24.2) | 0 (0) | 0 (0) | 230 (32.0) | 31 (16.8) | 19 (9.4) |  |
| Concentric hypertrophy | 299 (25.8) | 0 (0) | 0 (0) | 195 (27.2) | 56 (30.3) | 48 (23.6) |  |
| Eccentric hypertrophy | 212 (18.3) | 0 (0) | 0 (0) | 83 (11.6) | 20 (10.8) | 109 (53.7) |  |
| Chamber enlargement, n (%) | 173 (14.9) | 0 (0) | 0 (0) | 61 (8.5) | 29 (15.7) | 83 (40.9) | <0.001 |
| Wall motion abnormalities, n (%) | 311 (26.8) | 0 (0) | 0 (0) | 193 (26.9) | 45 (24.3) | 73 (36.0) | <0.001 |
| Valvular heart disease, n (%) | 797 (68.8) | 0 (0) | 0 (0) | 466 (64.9) | 147 (79.5) | 184 (90.6) | <0.001 |
| Aortic stenosis, n (%) | 55 (4.7) | 0 (0) | 0 (0) | 31 (4.3) | 13 (7.0) | 11 (5.4) | <0.001 |
| Structural heart disease, n (%) | 1052 (90.8) | 0 (0) | 0 (0) | 671 (93.5) | 178 (96.2) | 203 (100) | <0.001 |
| Increased filling pressure, n (%) | 522 (45.0) | 0 (0) | 0 (0) | 350 (48.7) | 81 (43.8) | 91 (44.8) | <0.001 |
| Admission diagnosis, n (%) |  |  |  |  |  |  |  |
| Hypertension | 504 (43.5) | 0 (0) | 18 (66.7) | 294 (40.9) | 95 (51.4) | 97 (47.8) | <0.001 |
| Diabetes | 466 (40.2) | 0 (0) | 13 (48.1) | 264 (36.8) | 86 (46.5) | 103 (50.7) | <0.001 |
| Myocardial infarction, this admission | 221 (19.1) | 0 (0) | 7 (25.9) | 155 (21.6) | 19 (10.3) | 40 (19.7) | <0.001 |
| Myocardial infarction, prior admission | 99 (8.5) | 0 (0) | 4 (14.8) | 59 (8.2) | 12 (6.5) | 24 (11.8) | 0.10 |
| PAOD | 68 (5.9) | 0 (0) | 1 (3.7) | 38 (5.3) | 9 (4.9) | 20 (9.9) | 0.08 |
| Atherosclerotic cardiovascular disease | 356 (30.7) | 0 (0) | 13 (48.1) | 224 (31.2) | 47 (25.4) | 72 (35.5) | <0.001 |
| Arrhythmia | 304 (26.2) | 2 (7.7) | 2 (7.4) | 166 (23.1) | 70 (37.8) | 64 (31.5) | <0.001 |
| Pneumonia | 351 (30.3) | 7 (26.9) | 7 (25.9) | 205 (28.6) | 75 (40.5) | 57 (28.1) | 0.03 |
| COPD | 69 (6.0) | 0 (0) | 0 (0) | 38 (5.3) | 13 (7.0) | 18 (8.9) | 0.12 |
| Cirrhosis | 62 (5.3) | 5 (19.2) | 2 (7.4) | 39 (5.4) | 8 (4.3) | 8 (3.9) | 0.02 |
| Dialysis | 265 (22.9) | 3 (11.5) | 3 (11.1) | 153 (21.3) | 44 (23.8) | 62 (30.5) | 0.02 |
| Stroke | 72 (6.2) | 0 (0) | 5 (18.5) | 33 (4.6) | 17 (9.2) | 17 (8.4) | 0.003 |
| Baseline evidence of motor, cognitive, or functional deficits | 390 (33.6) | 4 (15.4) | 11 (40.7) | 237 (33.0) | 71 (38.4) | 67 (33.0) | 0.16 |
| Bacteremia | 106 (9.1) | 3 (11.5) | 3 (11.1) | 73 (10.2) | 14 (7.6) | 13 (6.4) | 0.46 |
| Metastatic cancer or any blood-borne malignancy | 200 (17.3) | 14 (53.8) | 12 (44.4) | 147 (20.5) | 13 (7.0) | 14 (6.9) | <0.001 |
| Obesity | 183 (15.8) | 0 (0) | 7 (25.9) | 116 (16.2) | 27 (14.6) | 33 (16.3) | 0.12 |
| Pre-arrest events, n (%) |  |  |  |  |  |  |  |
| Hypotension | 301 (26.0) | 8 (30.8) | 4 (14.8) | 175 (24.4) | 51 (27.6) | 63 (31.0) | 0.20 |
| Respiratory insufficiency | 780 (67.3) | 14 (53.8) | 22 (81.5) | 472 (65.7) | 133 (71.9) | 139 (68.5) | 0.12 |
| Renal insufficiency | 550 (47.5) | 9 (34.6) | 12 (44.4) | 306 (42.6) | 102 (55.1) | 121 (59.6) | <0.001 |
| Hepatic insufficiency | 164 (14.2) | 7 (26.9) | 3 (11.1) | 104 (14.5) | 24 (13.0) | 26 (12.8) | 0.37 |
| Metabolic or electrolyte abnormality | 190 (16.4) | 5 (19.2) | 3 (11.1) | 115 (16.0) | 35 (18.9) | 32 (15.8) | 0.79 |
| Favorable neurological status 24 h before cardiac arrest | 531 (45.8) | 12 (46.2) | 8 (29.6) | 338 (47.1) | 75 (40.5) | 98 (48.3) | 0.21 |
| Peri-CPR conditions, n (%) |  |  |  |  |  |  |  |
| Arrest at night, n (%) | 352 (30.4) | 13 (50.0) | 10 (37.0) | 211 (29.4) | 51 (27.6) | 67 (33.0) | 0.14 |
| Arrest on weekend, n (%) | 256 (22.1) | 7 (26.9) | 8 (29.6) | 168 (23.4) | 37 (20.0) | 36 (17.7) | 0.32 |
| Arrest location, n (%) |  |  |  |  |  |  | 0.36 |
| Intensive care unit | 508 (43.8) | 12 (46.2) | 12 (44.4) | 303 (42.2) | 82 (44.3) | 99 (48.8) |  |
| General ward | 517 (44.6) | 14 (53.8) | 14 (51.9) | 328 (45.7) | 82 (44.3) | 79 (38.9) |  |
| Others | 134 (11.6) | 0 (0) | 1 (3.7) | 87 (12.1) | 21 (11.4) | 25 (12.3) |  |
| Monitoring, n (%) | 755 (65.1) | 17 (65.4) | 14 (51.9) | 453 (63.1) | 118 (63.8) | 153 (75.4) | 0.01 |
| Witnessed arrest, n (%) | 821 (70.8) | 18 (69.2) | 20 (74.1) | 509 (70.9) | 127 (68.6) | 147 (72.4) | 0.93 |
| Shockable rhythms, n (%) | 254 (21.9) | 5 (19.2) | 2 (7.4) | 140 (19.5) | 38 (20.5) | 69 (34.0) | <0.001 |
| Critical care interventions in place at time of arrest, n (%) |  |  |  |  |  |  |  |
| Mechanical ventilation | 277 (23.9) | 7 (26.9) | 5 (18.5) | 173 (24.1) | 45 (24.3) | 47 (23.2) | 0.96 |
| Antiarrhythmics | 198 (17.1) | 1 (3.8) | 3 (11.1) | 123 (17.1) | 35 (18.9) | 36 (17.7) | 0.36 |
| Vasopressors | 459 (39.6) | 12 (46.2) | 12 (44.4) | 267 (37.2) | 77 (41.6) | 91 (44.8) | 0.28 |
| Dialysis | 99 (8.5) | 1 (3.8) | 1 (3.7) | 50 (7.0) | 22 (11.9) | 25 (12.3) | 0.04 |
| Pulmonary artery catheter | 9 (0.8) | 0 (0) | 0 (0) | 5 (0.7) | 1 (0.5) | 3 (1.5) | 0.75 |
| Intra-aortic balloon pumping | 22 (1.9) | 0 (0) | 0 (0) | 16 (2.2) | 1 (0.5) | 5 (2.5) | 0.46 |
| CPR duration, min (IQR) | 21.0 (31.0) | 25.0 (32.3) | 30.0 (58.5) | 20.0 (30.8) | 21.0 (32.0) | 23.0 (28.0) | 0.36 |
| Post-ROSC interventions, n (%) |  |  |  |  |  |  |  |
| Extracorporeal membrane oxygenation | 185 (16.0) | 2 (7.7) | 2 (7.4) | 109 (15.2) | 23 (12.4) | 49 (24.1) | 0.005 |
| Therapeutic hypothermia | 30 (2.6) | 1 (3.8) | 1 (3.7) | 21 (2.9) | 3 (1.6) | 4 (2.0) | 0.81 |
| Percutaneous coronary intervention | 90 (7.8) | 1 (3.8) | 1 (3.7) | 57 (7.9) | 9 (4.9) | 22 (10.8) | 0.20 |
| Outcome, n (%) |  |  |  |  |  |  |  |
| Favorable neurological outcome at hospital discharge | 161 (13.9) | 2 (7.7) | 1 (3.7) | 106 (14.8) | 27 (14.6) | 25 (12.3) | 0.39 |
| Survival to hospital discharge | 275 (23.7) | 3 (11.5) | 2 (7.4) | 178 (24.8) | 50 (27.0) | 42 (20.7) | 0.07 |

COPD: chronic obstructive pulmonary disease; CPR: cardiopulmonary resuscitation; E/e’: ratio between early mitral inflow velocity and mitral annular early diastolic velocity; HF: heart failure; HFmrEF: heart failure with mildly reduced ejection fraction; HFpEF: heart failure with preserved ejection fraction; HFrEF: heart failure with reduced ejection fraction; IHCA: in-hospital cardiac arrest; IQR: interquartile range; LVEF: left ventricular ejection fraction; LVMI: left ventricular mass index; NT-proBNP: N-terminal pro-B natriuretic peptide; PAOD: peripheral arterial occlusive disease; ROSC: return of spontaneous circulation; TR: tricuspid regurgitation

**Supplemental Table 5.** Multivariable logistic regression model for primary and secondary outcomes in the sensitivity analysis

| Independent variable | Odds ratio | | 95% confidence interval | | | *p* value |
| --- | --- | --- | --- | --- | --- | --- |
| *Primary outcome: favorable neurological status at hospital discharge* | | | | | | |
| Concentric remodeling | 1.66 | | 1.03-2.69 | | | 0.04 |
| Age between 30 and 75 (y) | 2.08 | | 1.33-3.26 | | | 0.001 |
| Pneumonia | 0.28 | | 0.16-0.50 | | | <0.001 |
| Cirrhosis | 0.12 | | 0.02-0.90 | | | 0.04 |
| Dialysis | | 0.41 | | 0.23-0.73 | 0.002 | |
| Baseline evidence of motor, cognitive, or functional deficits | 0.32 | | 0.19-0.53 | | | <0.001 |
| Metastatic cancer or any blood borne malignancy | 0.24 | | 0.12-0.51 | | | <0.001 |
| Obesity | 0.55 | | 0.30-0.99 | | | 0.04 |
| Favorable neurological status 24 h before cardiac arrest | 3.39 | | 2.17-5.30 | | | <0.001 |
| Arrest in locations other than intensive care unit or general ward | 2.65 | | 1.54-4.55 | | | <0.001 |
| Monitoring | 1.55 | | 1.08-2.25 | | | 0.02 |
| Shockable rhythm | 2.36 | | 1.49-3.73 | | | <0.001 |
| Mechanical ventilation in place at time of arrest | 0.42 | | 0.22-0.80 | | | 0.009 |
| Antiarrhythmics in place at time of arrest | 2.03 | | 1.07-3.85 | | | 0.03 |
| Pulmonary artery catheter in place at time of arrest | 23.70 | | 4.45-126.22 | | | <0.001 |
| CPR duration less than 20 (min) | 8.08 | | 4.96-13.24 | | | <0.001 |
| Post-ROSC PCI | 2.56 | | 1.37-4.81 | | | 0.003 |
| *Secondary outcome: survival to hospital discharge* | | | | | | |
| Concentric remodeling | 1.53 | | 1.07-2.67 | | | 0.02 |
| Age between 39 and 75 (y) | 1.41 | | 1.02-1.96 | | | 0.04 |
| Cirrhosis | 0.17 | | 0.04-0.73 | | | 0.02 |
| Baseline evidence of motor, cognitive, or functional deficits | 0.55 | | 0.39-0.79 | | | 0.001 |
| Metastatic cancer or any blood borne malignancy | 0.29 | | 0.17-0.50 | | | <0.001 |
| Respiratory insufficiency | 0.59 | | 0.42-0.82 | | | 0.001 |
| Renal insufficiency | 0.64 | | 0.46-0.89 | | | 0.008 |
| Hepatic insufficiency | 0.48 | | 0.25-0.90 | | | 0.02 |
| Arrest in locations other than intensive care unit or general ward | 1.69 | | 1.07-2.67 | | | 0.02 |
| Shockable rhythm | 2.02 | | 1.40-2.90 | | | <0.001 |
| Vasopressors in place at time of arrest | 0.59 | | 0.42-0.84 | | | 0.003 |
| Pulmonary artery catheter in place at time of arrest | 5.99 | | 1.37-26.08 | | | 0.02 |
| CPR duration <23 (min) | 6.75 | | 4.62-9.86 | | | <0.001 |
| Post-ROSC PCI | 2.86 | | 1.65-4.95 | | | <0.001 |

CPR: cardiopulmonary resuscitation; PCI: percutaneous coronary intervention; ROSC: return of spontaneous circulation
